# Supplementary material for: Intermittent suppressive posaconazole therapy is ineffective at mitigating cardiac and digestive tract pathologies in an experimental model of chronic Chagas disease
Source: Antimicrob Agents Chemother. 2025 May 5;69(6):e01786-24. doi: 10.1128/aac.01786-24 (PMC12135509; doi:10.1128/aac.01786-24)
Supplement: Supplemental material — Fig. S1 and S2. [file aac.01786-24-s0001.pdf]

(A)

Treatment	Tissues from which clones where isolated
non-treated	peritoneum, peritoneum, peritoneum
initial treatment	visceral fat, mesentery, visceral fat
monthly treatment	oesophagous, skeletal muscle, peritoneum
weekly	subcutaneous fat, peritoneum, mesentery

(B)

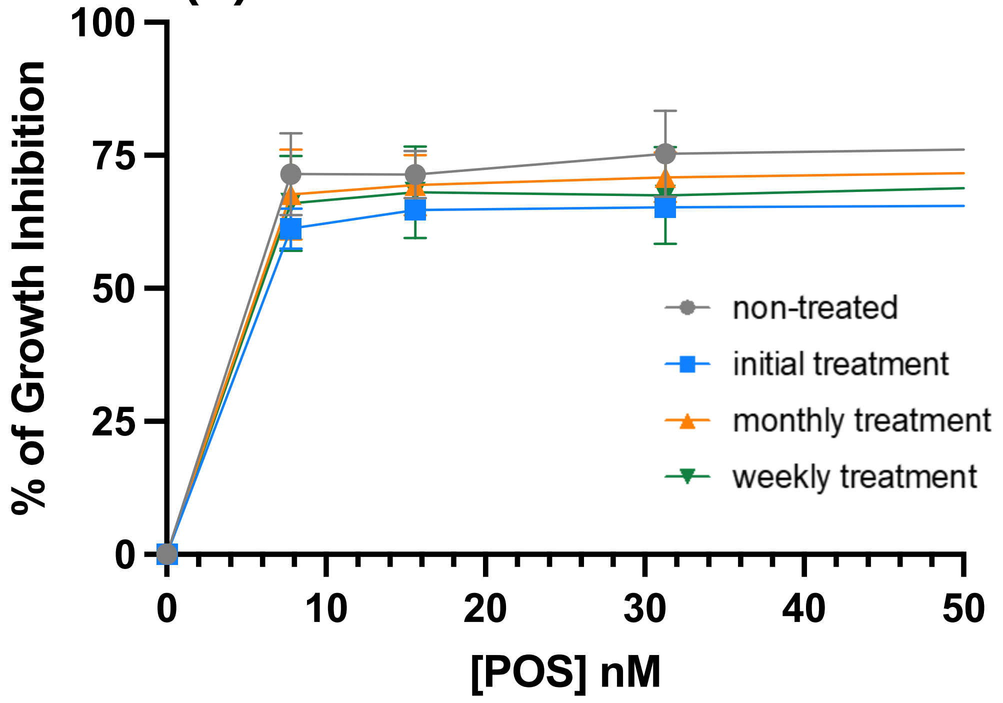

(C)

Dunnett's multiple comparisons test	*p*-value (one-way ANOVA)
non-treated vs. initial treatment	0.804
non-treated vs. monthly treatment	0.995
non-treated vs. weekly treatment	0.964

FIG S1. Posaconazole sensitivity of parasites isolated from mice at the experimental end-point. (A) Tissue source of parasites isolated from each of the mouse groups. (B) 8-point dose-response curves were used to assess posaconazole sensitivity, based on luminescence (Materials and Methods). Growth inhibition plateaued at 65-75%, and remained unaltered up to 500 mM. (C) No significant differences in posaconazole sensitivity were found between parasites isolated from non-treated or treated mice.

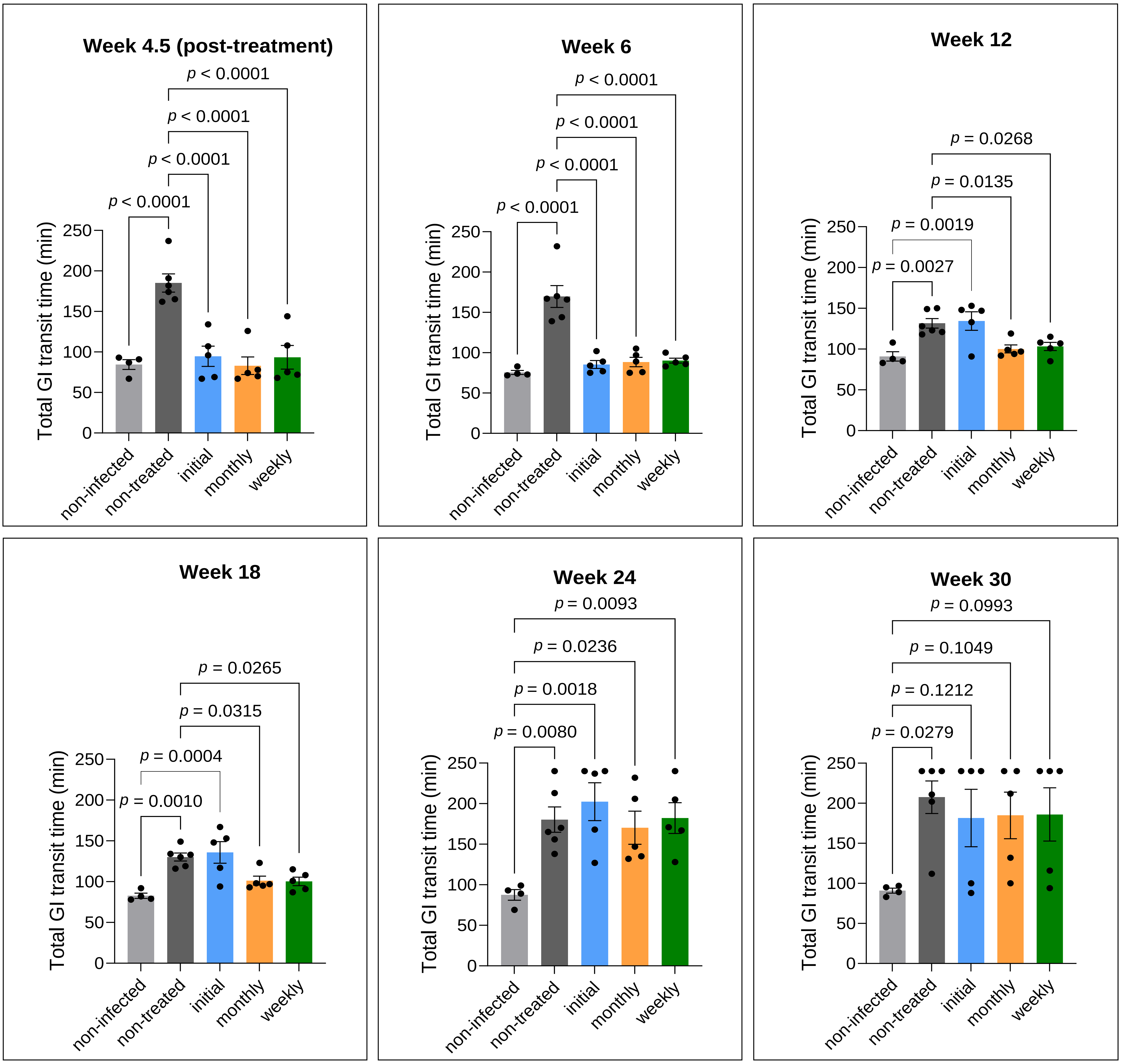

FIG S2. Statistical analysis of GI transit times between different groups. Uninfected C3H/HeN mice (n=4) and C3H/HeN mice were infected with the *T. cruzi* JR-Luc strain (non-treated, n=6; each treatment group, n=5). Each dot corresponds to a single mouse. Data of GI transit times are expressed as mean ± SEM, with all treated groups compared with non-treated and non-infected controls. A 4-hour (240 minutes) cut-off point for transit data acquisition was imposed for animal welfare reasons. Statistical analysis was carried out using ordinary one-way ANOVA, followed by Dunnett's multiple comparison post-hoc test, with *p*-values of significant differences ($p<0.05$).
